# Statistical inference with exchangeability and martingales

**DOI:** 10.1098/rsta.2022.0143

**Published:** 2023-05-15

**Authors:** Chris C. Holmes, Stephen G. Walker

**Affiliations:** ^1^ Department of Statistics, University of Oxford, Oxford, UK; ^2^ Department of Mathematics, University of Texas at Austin, Austin, TX, USA; ^3^ Department of Statistics & Scientific Computation, University of Texas at Austin, Austin, TX, USA

**Keywords:** bootstrap, parametric bootstrap, predictive inference, score function

## Abstract

In this paper, we start by reviewing exchangeability and its relevance to the Bayesian approach. We highlight the predictive nature of Bayesian models and the symmetry assumptions implied by beliefs of an underlying exchangeable sequence of observations. By taking a closer look at the Bayesian bootstrap, the parametric bootstrap of Efron and a version of Bayesian thinking about inference uncovered by Doob based on martingales, we introduce a parametric Bayesian bootstrap. Martingales play a fundamental role. Illustrations are presented as is the relevant theory.

This article is part of the theme issue ‘Bayesian inference: challenges, perspectives, and prospects’.

## 1. Introduction

Exchangeability forms one of the key starting points for the Bayesian approach, with many articles commencing along the lines ‘Assume the observations to beexchangeable’. The existence of *a prior* distribution, following such an assumption, has provided a foundation for the Bayesian paradigm (see ch. 4, Bernardo & Smith [1]). The existence of the prior from the representation theorem was established originally by de Finetti [2], for binary outcomes, and later for more general outcomes by Hewitt & Savage [3].

The exchangeable assumption on variables has the implication that the order of the labels becomes irrelevant. Consequently, $\left(x_{1:\infty }\right)$ forms an infinite exchangeable sequence if, and only if the joint distribution of $\left(x_{1},\ldots ,x_{n}\right)$ is invariant under permutations, i.e.

1.1 $$p(x_{1},\ldots ,x_{n})=p(x_{\sigma (1)},\ldots ,x_{\sigma (n)}),$$

for all permutations σ on {1,…,n} and for all n. Hewitt & Savage [3] referred to this as the joint density being symmetric, in contrast to de Finetti who talked about the exchangeability of the variables. Under the exchangeable assumption, de Finetti [2] showed, for {0,1} variables, that there is a representation of the joint density having the form

$$p(x_{1},\ldots ,x_{n})=\int \prod_{i=1}^{n}p(x_{i}|\theta )\;\Pi (d\theta ),$$

for some (prior) distribution function Π. For Bayesians, the prior forms the model, and so the sequence is set by the corresponding density function π(θ), assumed to exist, and defined on (0,1). Then $p(x|\theta )=\theta^{x}{\left(1-\theta \right)}^{1-x}$; i.e. the $\left(x_{i}\right)$ given θ are independent Bernoulli random variables. Hewitt & Savage [3] extended this result to more general types of symmetric density.

The existence of the prior π from the representation theorem was of great importance to the Bayesian argument. See, for example, Cifarelli & Regazzini [4] and Bernardo & Smith [1]. It motivated Bayesian analysis through the simple assumption of exchangeable observables. The breakthrough paper of Lindley & Smith [5] was based on the idea of exchangeability, as noted in the opening line from the abstract, '*The usual linear statistical model is reanalyzed using Bayesian methods and the concept of exchangeability*'.

Exchangeability is seen to be equivalent to saying that the order in which the data arrives makes no difference to inference; indeed, the probability model for the observations is symmetric. The predictive density follows under the usual conditioning process, i.e.

$$p(x_{n+1}|x_{1},\ldots ,x_{n})=\frac{p(x_{1},\ldots ,x_{n},x_{n+1})}{p(x_{1},\ldots ,x_{n})}.$$

Clearly, from the definition of exchangeability, the arrangement/ordering of the conditioned $\left(x_{1},\ldots ,x_{n}\right)$ becomes irrelevant.

It is also possible to view the predictive using an update of the prior π(θ), that is

1.2 $$p(x_{n+1}|x_{1},\ldots ,x_{n})=\int f(x_{n+1}|\theta )\;\pi (\theta |x_{1},\ldots ,x_{n})\;d\theta ,$$

where

1.3 $$\pi (\theta |x_{1},\ldots ,x_{n})=\frac{\prod_{i=1}^{n}f(x_{i}|\theta )\;\pi (\theta )}{p(x_{1},\ldots ,x_{n})},$$

is the posterior distribution, with $f(x_{i}|\theta )$ the normalized likelihood function for data point $\left(x_{i}\right)$.

Exchangeability has been viewed as a property on the data; creating a dependence structure where in reality there could be none. To see the argument, suppose the experimenter is to predict the first outcome $x_{1}$; in order to make a decision, for example. Write this predictive as $p_{0}(x)$. Once $x_{1}$ has been viewed, the experimenter might then wish to predict $x_{2}$. It would be an obstinate experimenter who persisted to predict with $p_{0}(x)$, since it would normally be assumed that witnessing $x_{1}$ would somehow revise the prediction for $x_{2}$. Indeed, the rationale for a statistical analysis and learning from data is that the observation $x_{1}$ provides information about the underlying process or population leading to improved prediction.

So write the revised predictive as $p_{x_{1}}(x)$. It is easy to see how such a procedure progresses; one eventually predicts $x_{n}$ using the revised density $p_{x_{1},\ldots ,x_{n-1}}(x)$. This results in a joint predictive density for the forthcoming observations to be

$$p(x_{1},\ldots ,x_{n})=p_{0}(x_{1})\;\prod_{i=2}^{n}p_{x_{1},\ldots ,x_{i-1}}(x_{i}).$$

If indeed the order is assumed not to matter; reflecting the notion that the observations could be independent and identically distributed (i.i.d.), then it would be required that $p(x_{1},\ldots ,x_{n})=p(x_{\sigma (1)},\ldots ,x_{\sigma (n)})$ be symmetric in the arguments and hence the existence of the prior-likelihood representation and the Bayesian approach. We write ‘could be’ here to reflect the notion that the data do not explicitly yield how they arose and the leap from the mathematics to the day to day practical data analysis relies on assumptions.

The above motivation de-mystifies Bayes. It is seen as a learning approach, updating beliefs as data arrive. It is no less and no more about the $p_{x_{1},\ldots ,x_{i}}(x)$ modelling a dependence between the observables arising as a consequence of revising predictives given observations. The crucial point is that it is the sequence of predictive distribution functions that depend on the observations rather than the next observation having some physical dependence on what has previously been seen. By physical dependence between variables x and y it would be assumed there is a form y=f(x) for some function f. In short, the exchangeable model is an appropriate learning model for i.i.d. data structures.

An early application of the predictive viewpoint for finite populations was developed in Roberts [6], Ericson [7] and later by Geisser [8,9]. This was extended to non-parametric predictive models in Lo [10] and Ghosh & Meeden [11]. Fortini *et al.* [12] explored concepts of predictive sufficiency and Fortini & Petrone [13,14] discuss the construction of a range of popular exchangeable non-parametric priors through a sequence of predictive distributions, motivated through a predictive de Finetti’s representation theorem.

There are a number of articles that focus on the construction of predictive distributions derived from representation theorems and relying on notions of exchangeability and partial exchangeability. Here, we mention Lijoi *et al.* [15,16], Favaro *et al.* [17] and De Blasi [18]. In such cases, and when the posterior is more complicated to derive, then full Bayesian inference can be achieved through these predictive distributions.

### (a) Prediction and model evidence

The predictive viewpoint is especially useful for understanding the plausibility of two or more competing theories (models) for the data, and the updating of such beliefs. We have seen how a hypothesis, such as a posited model M, for the data is characterized by the joint predictive $p_{M}(x_{1},\ldots ,x_{n})$, which provides an empirical model score when evaluated on the observed data, as seen through the 1-step predictions, and updates, on a sequence of held-out data

$$\log p_{M}(x_{1},\ldots ,x_{n})=\log p_{0}(x_{1})+\sum_{i=2}^{n}\log p_{x_{1:i-1}}(x_{i}).$$

Comparing two hypotheses (models), $p_{M_{1}}$ versus $p_{M_{2}}$, can be done by considering the Bayes factor (Kass & Raftery [19])

$$BF=\frac{p_{M_{1}}(x_{1},\ldots ,x_{n})}{p_{M_{2}}(x_{1},\ldots ,x_{n})},$$

obtained by the relative predictive scores on the observed sequence $x_{1:n}$. This highlights that Bayesian model choice is in essence a comparison of predictive performance on observables.

With exchangeability, the total score for any model is invariant to the n! possible ways of constructing the held-out predictive sequence

$$\log p_{M}(x_{1},\ldots ,x_{n})=\log p_{0}(x_{\sigma (1)})+\sum_{i=2}^{n}\log p_{\sigma (x_{1:i-1})}(x_{\sigma (i)}),$$

noting the kth element in the sum on the right-hand side has the form $p(x_{\sigma (k)}|x_{\sigma (1:k-1)})$. It is then straightforward to show [20,21] that the predictive score is equivalent to exhaustive cross-validation averaged over all n-choose-k possible held-out datasets of size k, for all k=1,…,n−1, when using the log predictive, $\log p(x|x_{1:i})$, as the cross-validation scoring rule. Moreover, Fong & Holmes [20] show this to be the unique scoring rule that ensures order coherency in the model evaluation, and this is irrespective of whether the model is true. Taken together, this highlights the usefulness of the predictive viewpoint and its focus on observables.

### (b) Generalizations

A concern could well be to what extent does a representation theorem cover different and more complex data structures than i.i.d. sequences, such as stationary sequences. A sequence $x_{1},x_{2},\ldots$ is stationary if

$$p_{x_{1},\ldots ,x_{n}}(y_{1},\ldots ,y_{n})=p_{x_{1+k},\ldots ,x_{n+k}}(y_{1},\ldots ,y_{n}),$$

for any n and any k. The representation of a stationary sequence due to Mitra [22] establishes the existence of a prior π(θ) for which it is possible to write

$$p(x_{1},\ldots ,x_{n})=\int \prod_{i=1}^{n}p(x_{i}|x_{1},\ldots ,x_{i-1},\theta )\;\pi (\theta )\;d\theta .$$

See also Aldous [23] and Lijoi *et al.* [15,16]. The connection between stationary sequences and exchangeable sequences has been considered by Berti *et al.* [24], where the link involves conditionally identically distributed (c.i.d.) sequences. A c.i.d. sequence is such that

$$p_{x_{n+k}}(x|x_{1},\ldots ,x_{n-1})=p_{x_{n}}(x|x_{1},\ldots ,x_{n-1}),$$

for all k≥1. That is, marginally, $x_{n+k}$ has the same distribution as $x_{n}$ for any k≥1.

For more complex data structures, the typical case in the modern data science era, there will be no representation theorem. Hence, some guiding principles are needed. The usual approach is to assign prior distributions to parameters of a likelihood function; to see Bayes as nothing more than likelihood times prior; see Robert [25]. While this is a simple plan, it is not motivated in a de Finetti sense. It is unclear that statistical uncertainty is being reflected in any meaningful way if the majority of prior distributions are constructed for convenience and attempted to be set up for objective reasons.

One attempt to cover more types of data structures is to use the notion of partial exchangeability. See for example, Camerlenghi *et al.* [26] who look at the problem from a predictive perspective. An interesting question is to what extent partial exchangeability extends to real data, such as covariate-driven non-homogeneous Markov time series with unknown order.

## 2. A new look at the Bayesian approach

Fong *et al.* [27] describe how Bayesian uncertainty can be interpreted through missing data. To make this notion concrete, in the case of i.i.d. observations from an infinite population, the missing data when a sample of size n has been observed; i.e. $x_{1:n}$, is the $x_{n+1:\infty }$. Our interpretation of the Bayesian approach is to deal with this uncertainty by providing a conditional distribution for $p(x_{n+1:\infty }|x_{1:n})$. More generally, the assumption behind most, if not all, statistical problems is that there is an amount of data, let us label it as $x_{\mathrm{comp}}$, which if observed, would yield the problem solved. That is, the parameter to be estimated is done so exactly, or the decision to be made can be done so correctly. For example, in a clinical trial if the recruited population was so large that any resulting uncertainty of the treatment effect estimate was considered negligible. So for an observed dataset, say $x_{\mathrm{obs}}$, the Bayesian approach is to construct a predictive distribution $p(x_{\mathrm{mis}}|x_{\mathrm{obs}})$ such that $x_{\mathrm{comp}}=(x_{\mathrm{mis}},x_{\mathrm{obs}})$.

The traditional Bayesian approach can be understood from this perspective. To illustrate this, let us return to the i.i.d. observations and for any sample of size n, there is a predictive density as in (1.2) and derived from the posterior (1.3). Then

$$p(x_{n+1:\infty }|x_{1:n})=\prod_{i=n+1}^{\infty }p(x_{i}|x_{1},\ldots ,x_{i-1}).$$

Now use this predictive to generate a large sample $\left(x_{n+1:N},x_{1:n}\right)$ which we will regard as $x_{\mathrm{comp}}$ for some suitably large N. Once this has been obtained, we define $\theta_{N}=\theta (x_{1:N})$, which is some functional of the data of interest. For example, it could be the sample mean. Doob [28], in his paper on the applications of martingales, establishes under extremely mild conditions, that if $\theta_{N}$ represents the posterior mean, then $\theta_{N}\to \theta$ a.s. and θ is a sample from the posterior derived from the sample of size n.

A less rigorous but intuitive construction is by noting the conventional Bayesian posterior on parameters of interest can be written as

$$\pi (\theta |x_{\mathrm{obs}})=\int \pi (\theta ,x_{\mathrm{mis}}|x_{\mathrm{obs}})\;dx_{\mathrm{mis}},$$

leading to

2.1 $$\pi (\theta |x_{\mathrm{obs}})=\int \pi (\theta |x_{\mathrm{comp}})\;p(x_{\mathrm{mis}}|x_{\mathrm{obs}})\;dx_{\mathrm{mis}},$$

where $x_{\mathrm{comp}}$ can be taken so large so as to yield no relevant uncertainty in the parameter of interest, θ, meaning in the context of a particular analysis for all practical purposes the conditional posterior can be replaced with a point estimate $\pi (\theta |x_{\mathrm{comp}})=1_{\theta (x_{\mathrm{comp}})}$. This highlights the source of the uncertainty in the Bayesian posterior, i.e. the left-hand side of (2.1), which arises from the missing data in the population. The conventional Bayesian posterior is recovered by taking $\theta (x_{\mathrm{comp}})$ as the posterior mean. A numerical solution to (2.1) follows a data-augmentation Monte Carlo approach by simulating $x_{\mathrm{mis}}∼p(x_{\mathrm{mis}}|x_{\mathrm{obs}})$ using the sequence of 1-step predictives and then picking off the resulting estimate $\theta (x_{\mathrm{comp}})$. As shown in Fong *et al.* [27], the computational solution is trivially parallel and often much faster to run than Markov chain Monte Carlo (MCMC). This missing data view also points to extensions of traditional Bayes involving more general predictive machines for use in $p(x_{\mathrm{mis}}|x_{\mathrm{obs}})$.

The Bayes posterior (2.1) has two components,

$$\pi (\theta |x_{\mathrm{obs}})=\int \underset{\text{Bayes estimate (large sample) }}{\underbrace{\pi (\theta |x_{\mathrm{comp}})}}\;\underset{\text{predictive (missing info.) }}{\underbrace{p(x_{\mathrm{mis}}|x_{\mathrm{obs}})}}\;dx_{\mathrm{mis}}.$$

The usual Bayesian approach is to construct the predictive, $p(x_{\mathrm{mis}}|x_{\mathrm{obs}})$, using a likelihood-prior mixture (1.2), that is fixed *a priori*, with inference focused on parameters of the likelihood. This ensures exchangeability and provides for a simple update via Bayes rule. The approach in Fong *et al.* [27] supports the replacement of $\pi (\theta |x_{\mathrm{comp}})$ with an estimate, $\theta (x_{\mathrm{comp}})$, using an appropriate functional targeting any statistic of interest, not necessarily indexing a likelihood function, and a predictive $p(x_{\mathrm{mis}}|x_{\mathrm{obs}})$ built using all available information at time of inference, not necessarily involving a prior. For example, in this regard, it is perfectly valid and reasonable to consider uncertainty in estimates arising from the Bayes linear model (e.g. Lindley & Smith [5]) obtained in the limit of large data, without assuming that the true data generating process is a linear model. The conventional Bayesian approach conflates these two issues, that the estimate refers to parameters in the predictive model assumed to be true (see §3).

### (a) Martingales

In fact, martingales are important for this view of the Bayesian methodology. Consider the posterior mean conditional on $x_{1:n+1}$, i.e.

$$\theta_{n+1}=\int \theta \;\pi (\theta |x_{1:n+1})\;d\theta =\frac{\int \theta \;f(x_{n+1}|\theta )\;\pi (\theta |x_{1:n})\;d\theta }{p(x_{n+1}|x_{1:n})},$$

where $x_{n+1}$ has been taken from the predictive $p(x_{n+1}|x_{1:n})$. Then, clearly, $E\;(\theta_{n+1}|x_{1:n})=\theta_{n}$ and so for m>n, the sequence of posterior means forms a martingale. Indeed, any mean functional would form a martingale as would the sequence of posterior distributions itself. Martingales are the important feature to ensure convergence of the parameter of interest.

Our argument, and promoted in Fong *et al.* [27], is that it is the martingale property which should be the determining criterion for the choice of $p(x_{n+1:\infty }|x_{1:n})$ and, more generally, for $p(x_{\mathrm{mis}}|x_{\mathrm{obs}})$. That is, if we define $\theta_{m+1}=\theta (x_{1:m+1})$ and $x_{m+1}$ has been taken from a ‘predictive’ $p(x_{m+1}|x_{1:m})$, then we require that $E(\theta_{m+1}|x_{1:m})=\theta_{m}$.

Fong *et al.* [27] focus on non-parametric models including a sequence of non-parametric predictives using copulas. Specifically, they take

2.2 $$P_{m+1}(x)=(1-a_{m})P_{m}(x)+a_{m}\;H(P_{m}(x),P_{m}(x_{m+1})),$$

where the weights are of the form $a_{m}=1/(a+m)\to 0$ and H is a partial derivative of a Gaussian copula function with correlation parameter ρ. That is $H(u,v)=\partial C_{\rho }(u,v)/\partial v$, which yields

$$H(u,v)=\Phi \left(\frac{\Phi^{-1}(u)-\rho \Phi^{-1}(v)}{\sqrt{1-\rho^{2}}}\right).$$

The sequence ${\left(P_{m}\right)}_{m>n}$ forms a martingale when the $x_{m+1}$ comes from $P_{m}(\cdot )$. The sequence commences at the distribution estimator $P_{n}$ from the observed sample $x_{1:n}$. Convergence of $\left(P_{m}\right)$ to a random distribution function follows from Berti *et al.* [29]. The limit $P_{\infty }$ is regarded as a random draw from the martingale posterior distribution.

The use of copulas here should not come as any great surprise. Consider the Bayesian predictive density given by

$$p(x|x_{1:n+1})=\frac{\int f(x|\theta )\;f(x_{n+1}|\theta )\pi (\theta |x_{1:n})\;d\theta }{p(x_{n+1}|x_{1:n})},$$

which can be written as

$$p(x|x_{1:n+1})=p(x|x_{1:n})\;\frac{\int f(x|\theta )\;f(x_{n+1}|\theta )\pi (\theta |x_{1:n})\;d\theta }{p(x|x_{1:n})\;p(x_{n+1}|x_{1:n})}.$$

The fraction part on the right is a copula density function, which can be written as

$$c_{n}(P_{n}(x)\;P_{n}(x_{n+1})),$$

for some copula density $c_{n}(\cdot ,\cdot )$. In most cases, this copula itself depends on the $x_{1:n}$; though in some conjugate Bayesian models, it only depends on the sample size n. Indeed, the complexity of prior to posterior procedures can be rendered difficult due to the nature of an intractable copula when it does depend on $x_{1:n}$. This motivated Hahn *et al.* [30] to adopt copula densities which only depended on the sample size and took the non-parametric copula distribution function

$$C(u,v)=(1-a_{m})\;uv+a_{m}\;C_{\rho }(u,v),$$

i.e. a sample size weighted combination of the independence copula and the Gaussian copula with correlation parameter ρ. The non-parametric credentials are valid here since the Dirichlet process sequence of predictives arise when, instead of $C_{\rho }$, we use the maximum copula, $\tilde{C}(u,v)=\min\{u,v\}$, which arises as ρ→1.

In order to better understand the martingale posterior and to investigate the parametric counterparts, we reconsider the bootstrap techniques, notably the bootstrap and Bayesian bootstrap. This will help us better understand the separate dealings with statistical uncertainty.

## 3. The bootstrap and Bayesian bootstrap

The bootstraps are very insightful tools with which to understand how the frequentist and Bayesian approaches to dealing with uncertainty differ. Both start with the empirical distribution function $P_{n}$, which puts mass 1/n at each of the observed data points.

The frequentist regards the sample of size n; i.e. $x_{1:n}$ as being the source of uncertainty, in that it is but one of many possible observed samples of size n. The uncertainty in this sample, and the consequences of this, could be ascertained if multiple independent samples of size n could be gathered. This is not possible as the true distribution is unknown; but the empirical distribution function is available. Sampling alternative datasets of size n is therefore taken from the empirical distribution function $P_{n}$. Multiple bootstrap samples can be obtained, $(x_{1:n}^{\left(b\right)})∼P_{n}$ for b=1,…,B. From each of these bootstrap samples, a statistic of interest can be computed, $T^{\left(b\right)}=T(x_{1:n}^{\left(b\right)})$ and consequently the sampling variance of $T=T(x_{1:n})$ can be estimated.

The Bayesian bootstrap was introduced in Rubin [31] and was seen as a Bayesian version of the bootstrap introduced in Efron [32]. The reason why this comparison was made is because both generate random distributions of the form

$$P=\sum_{i=1}^{n}q_{i}\;1_{x_{i}}.$$

For the Efron bootstrap, the random weights $\left(q_{i}\right)$ are based on multi-nomial sampling; q=Mn(n;1/n,…,1/n)/n. On the other hand, the Bayesian credentials for the Bayesian bootstrap is due to its connection with the Dirichlet process [33]. The Bayesian bootstrap generates random probability distributions which attach random weights to the set of observed data $x_{1:n}$; i.e.

3.1 $$P=\sum_{i=1}^{n}w_{i}\;1_{x_{i}},$$

where the $\left(w_{1:n}\right)$ have a Dirichlet distribution with all the parameters set to 1. This version of the bootstrap is very clear and that it belongs to the Bayesian non-parametric set of tools is also very clear. However, based on work done by Blackwell & MacQueen [34], which can be seen as a follow-up to the Doob [28] result, the Bayesian bootstrap can be viewed in an alternative and illuminating way.

Take a sample of size n and construct the empirical distribution function, $P_{n}$. Then sample $x_{n+1}∼P_{n}$ and update the empirical distribution with the new sample. That is, if $x_{n+1}$ is taken from $P_{n}$ then construct the new empirical predictive distribution $P_{n+1}$ given by

$$P_{n+1}=\frac{n\;P_{n}+1_{x_{n+1}}}{n+1}.$$

Continue in this fashion; to get $x_{n+1}$ and $P_{n+2}$ and so on. The sequence ${\left(P_{m}\right)}_{m>n}$ forms a martingale and some elementary theory for martingales on distribution functions [24] indicates that with probability one $P_{n}$ converges to a random distribution $P_{\infty }$. Since $P_{\infty }$ can only be of the form (3.1) it is only a matter of finding the weights attached to the $\left(w_{i}\right)$. It is not difficult to establish the correct Dirichlet weights for them; see Blackwell & MacQueen [34] and Sethuraman [35].

Thus we see the Bayesian bootstrap is constructing a predictive distribution $P(x_{n+1:\infty }|x_{1:n})$ and it is easy to see the sequence is given by

3.2 $$P_{m+1}(x)=(1-a_{m})\;P_{m}(x)+a_{m}\;1(x_{m+1}\leq x),$$

with $x_{m+1}$ coming from $P_{m}$, and $a_{m}=1/(1+m)$. The sequence forms a martingale and this is easy to check. The limit $P_{\infty }$ can be sampled according to (3.1). Then (3.1) can be viewed as a ‘short-cut’ to avoid the sampling of the $\left(P_{m}\right)$. The non-trivial differences between the two types of bootstrap is quite clear.

Given $P_{\infty }$, and the missing data view from §2, we can proceed to make inference by picking off an estimate of any particular statistic of interest, $\theta (P_{\infty })$. One particularly interesting statistic is the estimate of the parameters in a statistical model, f(x∣θ), minimizing the self-information loss (log-likelihood), i.e.

$$\theta (P_{\infty })=\arg\min_{\theta }\left[\int -\log f(x|\theta )\;P_{\infty }(dx)\right].$$

With $P_{\infty }$ as the Bayesian bootstrap leads to the weighted-likelihood bootstrap and extensions thereof [36,–41]. As noted in §2, we see it as perfectly valid to think about the Bayesian uncertainty in the value of θ arising from a statistical model, f(x∣θ), fit in the limit of large data, without assuming that the data were generated by this statistical model for some unknown setting of θ.

It is to be noted that the sequence in (2.2) can be seen as a smoothed version of (3.2), where the indicator function has been smoothed to the Gaussian copula. Also note that as ρ→1 it is that $H_{\rho }(u,v)\to 1(u=v)$.

The frequentist is replicating statistics of sample size n. The Bayesian is obtaining ‘true’ statistics via $T=T(P_{\infty })$ and acknowledging the uncertainty in any single T(P) through the posterior it generates. It is this latter view of the Bayesian handling of uncertainty that is of interest to us. It is our intention to extend the family of Bayesian bootstraps and to use these to construct posterior distributions and adopting this as the fundamental Bayesian paradigm. That is, the construction of $p(x_{n+1:\infty }|x_{1:n})$, namely $p(x_{\mathrm{mis}}|x_{\mathrm{obs}})$, is the fundamental task while ensuring convergence of the statistics of interest, $\theta (x_{\mathrm{comp}})$, and the sequence of predictive distribution. This can be guaranteed via the use of martingales.

Our understanding of the construction of $p(x_{n+1:\infty }|x_{1:n})$ is that it is prior free; it starts at the sample size n. Instead of generating multiple replica datasets of size n from this, along the lines of the frequentist bootstrap, we use it to generate the missing data along the line of the Bayesian bootstrap and the construction of a posterior distribution. To acknowledge the fusion here,we label the final output as a form of *frequentist posterior*; as we can take $p(x_{n+1:\infty }|x_{1:n})$ to be the product of $f(x_{m+1}|{\hat{\theta }}_{m})$ for m=n,…,∞, where the ${\hat{\theta }}_{m}$ would be the maximum-likelihood estimator based on $x_{1:m}$.

## 4. A parametric Bayesian bootstrap

Consider the data model f(⋅∣θ). The parametric bootstrap introduced in Efron [42] replaces the empirical distribution function with the plug-in density estimator, $f(\cdot |\hat{\theta })$. The reasons for this become straightforward if the model is assumed to be correct, or is approximately so. Here, the $\hat{\theta }=\theta (x_{1:n})$ is some functional of the data, such as the mle. In all other respects, the parametric bootstrap mirrors that of the non-parametric one. So a bootstrap sample $\left(x_{1:n}^{\left(b\right)}\right)$ is taken i.i.d. from $f(\cdot |\hat{\theta })$. For each sample, a new mle estimator is obtained as ${\hat{\theta }}^{\left(b\right)}$ and the collection of such can be used to estimate the sampling distribution and specifically the variance of $\hat{\theta }$.

Efron observed that the distribution of the $\left({\hat{\theta }}^{\left(b\right)}\right)$ resembles closely a possible posterior distribution and advocated it could be used in some importance sampling procedure to obtain samples from a posterior. It cannot be regarded as a posterior itself as it is not derivable from *a prior* and the more important point is that it is a distribution for the estimator, not for the true parameter value.

The connection and differences between the bootstrap and Bayesian bootstrap have shown us that a different sampling strategy from $f(\cdot |\hat{\theta })$ implements our Bayesian approach. The Bayesian version assumes further the $\hat{\theta }$ is an unbiased estimator for the true value θ. We then sample $x_{n+1}$ from the data model using the plug-in $\hat{\theta }$ which is then updated to ${\hat{\theta }}_{n+1}$ using $x_{n+1}$, and so on. In short, given $x_{1:m}$ with estimator ${\hat{\theta }}_{m}$, we perform

$$x_{m+1}∼f(\cdot |{\hat{\theta }}_{m})\;\text{and}\;{\hat{\theta }}_{m+1}=\theta (x_{1:m+1}),\;m\geq n.$$

The $\left({\hat{\theta }}_{m\geq n}\right)$ form a martingale and provided the variances do not grow but converge then a random $\theta_{\infty }$ exists and can be seen as a sample from a ‘parametric martingale’ posterior distribution $\pi (\cdot |x_{1:n})$.

It is worth at this point considering a simple example. Suppose we have a normal location parameter model for which the data model is $\text{N}(\cdot |\theta ,\sigma^{2})$ where θ is unknown and σ is known. Then from a sample of size n we get the ${\hat{\theta }}_{n}={\bar{x}}_{n}$ which is an unbiased estimator for θ. We then sample $x_{n+1}$ from $\text{N}(\cdot |{\bar{x}}_{n},\sigma^{2})$ and get

$${\hat{\theta }}_{n+1}={\bar{x}}_{n+1}=\frac{n{\hat{\theta }}_{n}+x_{n+1}}{n+1},$$

and so on. This is a martingale for ${\left(\hat{\theta }\right)}_{m>n}$ and to investigate convergence, we consider the $\tau_{m}^{2}=\text{Var}\;{\hat{\theta }}_{m}$. Now writing ${\hat{\theta }}_{m+1}={\hat{\theta }}_{m}+\sigma \;z/(m+1)$, where z is a standard normal random variable, we get

$$\tau_{m+1}^{2}=\tau_{m}^{2}+\frac{\sigma^{2}}{{\left(m+1\right)}^{2}}⟹\tau_{m+1}^{2}=\sigma^{2}\sum_{k=n+1}^{m}\frac{1}{k^{2}},$$

resulting in ${\hat{\theta }}_{\infty }$ being a normal random variable with mean ${\hat{\theta }}_{n}$ and variance $c_{n}\;\sigma^{2}$ where $c_{n}=\sum_{k>n}1/k^{2}\approx 1/n$. This leads to the frequentist posterior as a probability measure directly on the unknown ${\hat{\theta }}_{\infty }$ as approximately $N({\hat{\theta }}_{n},\sigma^{2}/n)$.

In general, we assume we can write

$${\hat{\theta }}_{m+1}={\hat{\theta }}_{m}+s_{m}(x_{m+1},{\hat{\theta }}_{m}),$$

for some function $s_{m}$, where $x_{m+1}$ comes from $f(\cdot |{\hat{\theta }}_{m})$. Hence, for the martingale, we require

$$E\;s_{m}(x_{m+1},{\hat{\theta }}_{m})=0,$$

and so the $s_{m}$ resembles or acts similarly to a score function. Note that it is easy to compute the variance of $\theta_{\infty }$ since it would be equal to

$$\tau_{\infty }^{2}=\sum_{k>n}\text{Var}\;s_{k-1}(x_{k},{\hat{\theta }}_{k-1}).$$

This finding motivates a potentially simpler idea, which is to use the score function from the data model; i.e. define s(x,θ)=∂log⁡f(x∣θ)/∂θ. Then we can use the sequence of estimators as

$$\theta_{m+1}=\theta_{m}+\epsilon_{m}\;s(x_{m+1},\theta_{m}),$$

where $x_{m+1}$ comes from $f(\cdot |\theta_{m})$. Here, $\epsilon_{m}$ would act as a step size and the updating here resembles a gradient descent algorithm. The variance of $\theta_{\infty }$ would now be

$$\tau_{\infty }^{2}=\sum_{k>n}\epsilon_{k-1}^{2}\;\text{Var}\;s(x_{k},\theta_{k-1}),$$

which if $\epsilon_{k}$ is of order 1/(k+n) then $\tau_{\infty }^{2}$ will be of order 1/n, which is usual for parametric posterior distributions.

The idea here for updating the parameter is as follows. If there is a procedure for updating the parameter estimator as real data arrive; e.g. the mle or gradient descent for more complex problems, then this procedure continues post data where the data are now replaced by sample data from the model using the current parameter estimate. This is done in such a way so that the ensuing sequence of parameter estimators forms a martingale.

Here, it is worth pointing out about the form for $p(x_{\mathrm{mis}}|x_{\mathrm{obs}})$, constructed sequentially. Unless the model is based on exchangeability, the ordering of the $x_{n+1:\infty }$ will matter. For example, without exchangeability, the joint density of $\left(x_{n+2},x_{n+1}\right)$ will not share the same density as $\left(x_{n+3},x_{n+2}\right)$. We are not aware of any practical consequence of this. As well, if the sequence is c.i.d. the proximity to an exchangeable sequence depends on the sample size and asymptotically a c.i.d. is exchangeable. On the other hand, if exchangeability is regarded as an essential component to Bayesian uncertainty quantification, then the prior to posterior will be the best choice.

## 5. Illustrations

**Example 5.1.**

The first and obvious example is the normal location parameter; the martingale here is $\theta_{m+1}=(m\theta_{m}+y_{m+1})/(m+1)$ for m≥n with $y_{m+1}∼\text{N}(\theta_{m},\sigma^{2})$. So $\theta_{m+1}=\theta_{m}+\sigma \;z_{m}/(m+1),$ which is based on the $\theta_{m}$ being the sample mean at each iteration. Here, the $\left(z_{m}\right)$ are independent standard normal random variables. The frequentist posterior is normal with mean $\theta_{n}$, the sample mean from the observed data, and with variance $\sigma_{n}^{2}=\sigma^{2}\sum_{m=n}^{\infty }1/{\left(m+1\right)}^{2}$. The sum is approximately 1/n.

If the variance is also unknown, we set up two martingales, one for the mean and one for the variance. Now we take the same martingale for the mean and take $y_{m+1}∼\text{N}(\theta_{m},\sigma_{m}^{2})$, where we take

$$\sigma_{m}^{2}=\frac{\sum_{i=1}^{m}{\left(y_{i}-\theta_{m}\right)}^{2}}{\kappa_{m}}\;\text{and}\;\kappa_{m}=\sum_{i=1}^{m}\frac{i}{i+1}.$$

The martingale for $\left(\sigma_{m}^{2}\right)$ is explained as

$$\kappa_{m+1}\sigma_{m+1}^{2}=\sum_{i=1}^{m}y_{i}^{2}+y_{m+1}^{2}-{\left(m\theta_{m}+y_{m+1}\right)}^{2}/(m+1),$$

which can be tidied up to give

$$\kappa_{m+1}\sigma_{m+1}^{2}=\kappa_{m}\sigma_{m}^{2}+\frac{m}{m+1}{\left(\theta_{m}-y_{m+1}\right)}^{2}.$$

Taking a conditional expectation keeping $y_{1:m}$ fixed,

$$\kappa_{m+1}E\;(\sigma_{m+1}^{2}|y_{1:m})=\sigma_{m}^{2}\kappa_{m+1},$$

which demonstrates the martingale.

**Figure 1.**
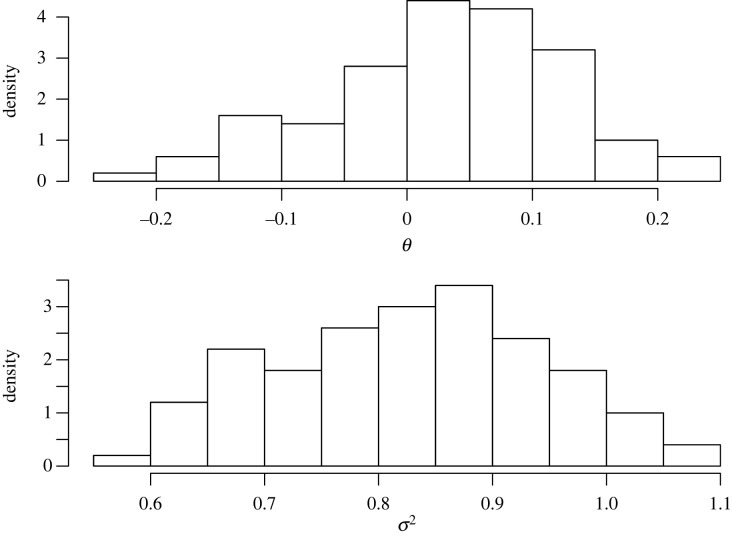
Marginal frequentist posterior distribution for θ and $\sigma^{2}$.

We take a worked illustration with n=100 independent samples from a standard normal distribution. The value of N we use to stop the martingales is 1000. This value appears large enough for the martingales to converge. A histogram of the 100 samples from the 2×100 martingales we ran are presented in figure 1. The sample mean and sample variance from the data were 0.048 and 0.848, respectively.

**Example 5.2.**

Here, we consider the Poisson model; with density function

$$f(y,\theta )=\theta^{y}/y!\;e^{-\theta },\;y\in \{0,1,2\ldots \}.$$

Hence, $l^{\prime }(y,\theta )=-1+y/\theta$ and $l^{\prime \prime }(y,\theta )=-y/\theta^{2}$. Taking $y_{m+1}∼\text{Pois}(\theta_{m})$, the martingale becomes

$$\theta_{m+1}=\theta_{m}+\frac{\left(y_{m+1}/\theta_{m}-1\right)}{\sum_{i=1}^{m}y_{i}/\theta_{m}^{2}}=\theta_{m}+(y_{m+1}-\theta_{m})/m.$$

In this case, it is easy to compute the limit values of the mean and variance. So $E\;\theta_{\infty }=\theta_{n}$, due to the martingale, and

$$\text{Var}\;\theta_{\infty }=\theta_{n}\;\sum_{m\geq n}{\frac{1}{m}}^{2}\approx \frac{\theta_{n}}{n}.$$

Note that the Bayesian objective posterior using the prior π(θ)∝1/θ is gamma with mean $\theta_{n}$ and variance $\theta_{n}/n$.

**Figure 2.**
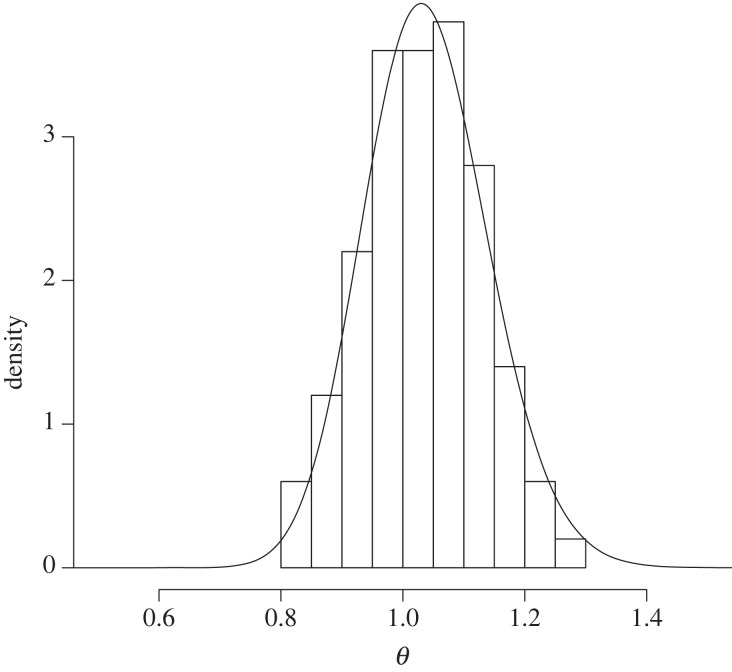
Frequentist posterior (histogram) from the Poisson example, alongside the Bayesian gamma objective posterior (line).

We ran an illustration using n=100 and taking independent samples from the Poisson distribution with mean 1. We collected 100 frequentist posterior samples by running 100 martingales each to a length of N=2000. The posterior is presented as the histogram and the objective Bayesian gamma posterior with prior π(θ)∝1/θ is shown alongside in figure 2.

**Example 5.3.**

In this part, we consider a two parameter gamma model, $f(y,\theta )=b^{a}y^{a-1}e^{-yb}/\Gamma (a)$. So l(y,a,b)=alog⁡b−log⁡Γ(a)+alog⁡y−by and so

$$\frac{\partial l}{\partial a}=\log b-\psi (a)+\log y\;\text{and}\;\frac{\partial l}{\partial b}=\frac{a}{b-y},$$

where ψ is the digamma function, with

$$\frac{\partial^{2}l}{\partial a^{2}}=-\psi^{\prime }(a),\;\partial^{2}l/\partial b^{2}=1/b^{2}\;\text{and}\;\frac{\partial^{2}l}{\partial a\partial b}=-\frac{a}{b^{2}}.$$

Defining the matrix and vector

$$H_{m}=m\left(\begin{array}{ll} \psi (a_{m}) & -1/b_{m}\\ -1/b_{m} & a_{m}/b_{m}^{2} \end{array}\right)\;\mathrm{and}\;v_{m}=\left(\begin{array}{l} \log b_{m}-\psi (a_{m})+\log y_{m+1}\\ a_{m}/b_{m}-y_{m+1} \end{array}\right),$$

where $y_{m+1}∼\text{Ga}(a_{m},b_{m})$. Then the martingale is $\theta_{m+1}=\theta_{m}+H_{m}^{-1}\;v_{m}.$

**Figure 3.**
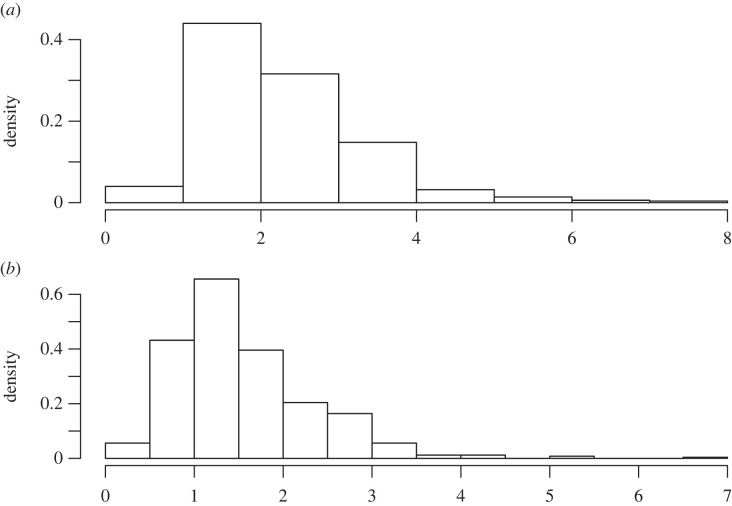
(*a* and *b*) Frequentist posteriors from the gamma example.

We took n=100 samples from the gamma distribution with a=3 and b=2 and 500 martingales were run each with a run length of 1000. The frequentist distribution for (a,b) is presented in figure 3.

**Example 5.4.**

This example looks at the standard linear regression model,

$$y_{i}=x_{i}^{\prime }\beta +\sigma \varepsilon_{i},\;i=1,\ldots ,n,$$

where $x_{i}$ is a p×1 vector of regression variables and β is a p×1 vector of unknown coefficients. We assume the σ is known and the $\left(\varepsilon_{i}\right)$ are independent standard normal errors. The estimator based on the observed sample of size n is $\beta_{n}={\left(X^{\prime }X\right)}^{-1}X^{\prime }y$ where X is the n×p matrix from ${\left(x_{i}\right)}_{i=1:n}$.

We construct the martingale using

$$\frac{\partial l}{\partial \beta_{j}}=\sum_{i=1}^{n}(y_{i}-x_{i}^{\prime }\beta )\;x_{ij}\;\text{and}\;\frac{\partial^{2}l}{\partial \beta_{j}\beta_{k}}=-\sum_{i=1}^{n}x_{ij}x_{ik},$$

where $l(\beta )=\frac{1}{2}\sum_{i=1:n}{\left(y_{i}-x_{i}^{\prime }\beta \right)}^{2}$. The martingale becomes, for m≥n,

$$\beta_{m+1}=\beta_{m}+{\left(\sum_{i=1}^{m}x_{i}\;x_{i}^{\prime }\right)}^{-1}\;x_{m+1}\;(y_{m+1}-x_{m+1}^{\prime }\beta_{m}),$$

where $y_{m+1}∼\text{N}(x_{m+1}^{\prime }\beta_{m},\sigma^{2})$. Here, the ${\left(x_{i}\right)}_{m>n}$ can be arbitrarily chosen or sampled from the observed values ${\left(x_{i}\right)}_{i=1:n}$. The clear choice would be to sample from the empirical distribution.

**Figure 4.**
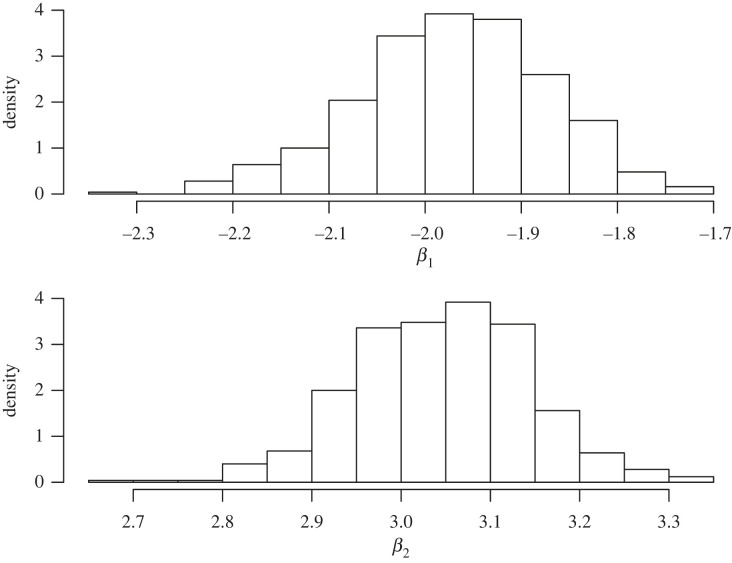
Frequentist posteriors from the linear model example.

For the specific illustration, we took n=100 samples with the true β=(−2,3) and all the $\left(x_{ij}\right)$ sampled independently from a standard normal distribution, with σ=1. We ran 500 martingales and each was run for a length of size 1000. The 500 frequentist posterior samples are presented in figure 4.

**Example 5.5.**

A further example is a Markov-driven time series with standard normal marginals. So

$$y_{i}=\rho \;y_{i-1}+\sqrt{1-\rho^{2}}\varepsilon_{i},\;i=2,\ldots ,n,$$

where the $\left(\varepsilon_{i}\right)$ are independent standard normal random variables. Then $\rho_{n}=\sum_{i=2}^{n}y_{i}\;y_{i-1}/(n-1)$. The log-density is given by

$$l(y,\rho )=-\frac{1}{2}\log(1-\rho^{2})-\frac{1}{2}{\left(y_{2}-\rho y_{1}\right)}^{2}/(1-\rho^{2}).$$

The first and second derivatives with respect to ρ are easy to obtain, e.g.

$$\frac{\partial l}{\partial \rho }=\frac{\rho }{\left(1-\rho^{2}\right)}-\frac{\rho {\left(y_{2}-\rho y_{1}\right)}^{2}}{\left(1-\rho^{2}\right)}+\frac{y_{1}(y_{2}-\rho y_{1})}{{\left(1-\rho^{2}\right)}^{2}}.$$

Hence, the martingale is

$$\rho_{m+1}=\rho_{m}-\frac{\partial l/\partial \rho (y_{m+1},\rho_{m})}{\sum_{i=1}^{m}\partial^{2}l/\partial \rho^{2}(y_{i},\rho_{m})},$$

where $y_{m+1}$ is normal with mean $\rho y_{m}$ and variance $1-\rho_{m}^{2}$.

**Figure 5.**
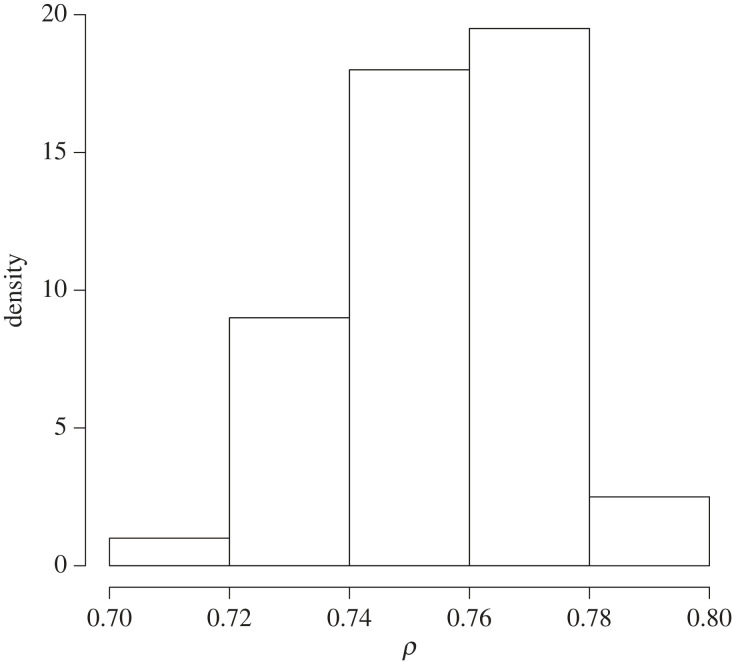
Frequentist posterior from the Markov time series model example.

By way of an illustration, we took $y_{1}=0$ and then the $\left(y_{i}\right)$ for i=1,…,n, with n=100, according to the Markov model with ρ=0.7. The estimator turned out to be 0.758. We ran 100 martingales and each was run for a length of 500 iterations. The histogram of the 100 samples from the frequentist posterior is provided in figure 5.

## 6. Theory

The theory we present here is about martingales and the convergence of such to limits. For general information on martingales, see Williams [43]. The first result is for the sequence of random distributions ${\left(P_{m}\right)}_{m>n}$, which form a martingale in the sense that

$$\text{E}(P_{m+1}(y)|y_{1:m})=P_{m}(y)\;\text{for all}\;y.$$

Since a probability distribution is bounded the $P_{m}(y)$ converges almost surely to a $P_{\infty }(y)$ for each y. That the sequence ${\left(P_{m}\right)}_{m=n+1}^{\infty }$ converges with probability one to a random distribution function is a consequence of theorem 2.2 appearing in Berti *et al.* [29]. A modified version of the theorem relevant to the present paper is provided here.

**Theorem 6.1.**

*Given* $P_{n}$, if $P_{m}(y)$, *is a sequence of random distribution functions, for* m>n, *and converges almost surely to* $P_{\infty }(y)$ *for each* y *with* $\text{E}\;P_{m}=P_{n}$, *and* $P_{n}$ *is a tight distribution function, then* $P_{m}$ *converges weakly to the random distribution function* $P_{\infty }$ *almost surely*.

From the construction of $P_{m}(y)$ for m>n, it is clearly evident that $P_{m}(y)$ is a martingale for each y. From the martingale convergence theorem, there exists a $P_{\infty }(y)$ for each y for which $P_{m}(y)\to P_{\infty }(y)$ almost surely. Now $P_{n}$ is a fixed distribution function and hence for any ϵ>0 there will be a compact set K such that $P_{n}(K)>1-\epsilon$. Hence, as such, $P_{n}$ is a tight distribution function. For more on tightness, see Billingsley [44]. Hence, from the theorem, $P_{i}$ converges weakly to the random distribution function $P_{\infty }$ almost surely.

For a general martingale for the parameter ${\left(\theta_{m}\right)}_{m>n}$, we have the following martingale convergence theorem:

**Theorem 6.2.**

*If* ${\left(\theta_{m}\right)}_{m>n}$ *is a martingale and* $\underset{m}{\sup}\text{E}|\theta_{m}{|}^{2}<\infty$ *then there exists a random variable* $\theta_{\infty }$ *such that* $\theta_{m}\to \theta_{\infty }$ *a.s. and* $\text{E}(\theta_{\infty })=\theta_{n}$.

*In practice, we consider* $\text{Var}(\theta_{m})$.

*One of the martingales on which we rely is the stochastic gradient descent, or ascent in our case, algorithm. If* $\left(y_{n}\right)$ *are i.i.d. from* $f(\cdot |\theta^{*})$ *and*

6.1 $$\theta_{n}=\theta_{n-1}+\sigma_{n}^{2}\;s(y_{n},\theta_{n-1}),$$

for some suitable sequence $\left(\sigma_{n}^{2}\right)$ *and starting point* $\theta_{0}$, where s(y,θ)=∂log⁡f(y∣θ)/∂θ, *then we require that* $\theta_{n}\to \theta^{*}$ *as* n→∞ *in the sense that* $E[{\left(\theta_{n}-\theta^{*}\right)}^{2}]\to 0$. *See, for example, Murphy* [45].

The essence of the argument for convergence is as follows:

$$\begin{array}{rl} {\left(\theta_{n+1}-\theta^{*}\right)}^{2} & \;={\left(\theta_{n}+\sigma_{n+1}^{2}\;s(y_{n+1},\theta_{n})-\theta^{*}\right)}^{2}\\ & \;={\left(\theta_{n}-\theta^{*}\right)}^{2}+\sigma_{n+1}^{4}s^{2}(y_{n+1},\theta_{n})+2\sigma_{n+1}^{2}s(y_{n+1},\theta_{n})\;(\theta_{n}-\theta^{*}). \end{array}$$

The last term on the right side is the important part and write the expectation as

$$2\sigma_{n+1}^{2}{\left(\theta_{n}-\theta^{*}\right)}^{2}\;\frac{s(\theta_{n},\theta^{*})}{\theta_{n}-\theta^{*}},$$

where

$$s(\theta ,\theta^{*})=\int s(y,\theta )\;f(y|\theta^{*})\;dy,$$

and the assumption is that for θ sufficiently close to $\theta^{*}$, it is that $s(\theta ,\theta^{*})>0$ if $\theta <\theta^{*}$, $s(\theta ,\theta^{*})<0$ if $\theta >\theta^{*}$ and $s(\theta^{*},\theta^{*})=0$, and further that $s(\theta ,\theta^{*})/(\theta -\theta^{*})<-L$ within a neighbourhood for some finite L>0. Hence, we can write

$$E\;[{\left(\theta_{n+1}-\theta^{*}\right)}^{2}|y_{1:n}]\leq {\left(\theta_{n}-\theta^{*}\right)}^{2}\{1-2L\sigma_{n+1}^{2}\}+\sigma_{n+1}^{4}s^{2}(y_{n+1},\theta_{n}).$$

Proceeding, for some constants $c_{1}>0$ and $c_{2}>0$, we have

$$d_{n+1}\leq (1-c_{1}\sigma_{n+1}^{2})\;d_{n}+c_{2}\sigma_{n+1}^{4},\;d_{n}=E\;[{\left(\theta_{n}-\theta^{*}\right)}^{2}],$$

and recall $\sigma_{n+1}^{2}$ is of order 1/n. It is now standard mathematics to demonstrate that $d_{n}\to 0$.

This supports (6.1) as a Bayesian style learning algorithm, updating the Bayesian estimator $\theta_{0}$ with data. Once we reach the end of the data, we revert to a martingale, generating the data with the current point estimator and proceeding with (6.1) to infinity. The $\theta_{\infty }$ exists and has expected value $\theta_{n}$ and the variance, just as with usual Bayesian updating, is O(1/n). Hence, the ‘posterior’ is available and which we regard as well motivated as the traditional Bayesian approach.

In order for the martingale to converge, we need to look at $\text{Var}(\theta_{m})$. Now if

6.2 $$\theta_{m+1}=\theta_{m}+\sigma_{m}^{2}\;s(y_{m+1},\theta_{m}),$$

then

$$\text{Var}(\theta_{m+1})=\text{Var}(\theta_{m})+\sigma_{m}^{4}\;\text{E}\;\text{Var}\{\;s(y_{m+1},\theta_{m})\},$$

where the variance applies to the $y_{m+1}$ given $\theta_{m}$ and the expectation with respect to $\theta_{m}$. Hence,

$$\text{Var}(\theta_{\infty })=\sum_{m>n}\sigma_{m}^{4}\;\text{E}\;\text{Var}\{\;s(y_{m+1},\theta_{m})\},$$

which we need to ensure is finite. This can be achieved through a focus on $\sigma_{m}$, the interpretation of which is to be found in (6.2) and illustrated in the examples we have presented, specifically how it is related to posterior variances involving the Fisher information.

## 7. Discussion

From the understanding of Bayesian inference through the construction of $p(x_{\mathrm{mis}}|x_{\mathrm{obs}})$ in order to obtain $x_{\mathrm{comp}}$ we relax the strict assumptions which take this predictive to start with a prior distribution on a parameter. We argue it is straightforward to construct such a predictive, using density estimators, for example, even based on maximum-likelihood estimators, which in the non-parametric case could be represented by the empirical distribution function.

A key to the construction of $p(x_{\mathrm{mis}}|x_{\mathrm{obs}})$ is the martingale, indeed, it is a key component of the Bayesian framework, though often concealed from view as the prior to posterior update puts it firmly into the background. The martingale ensures convergence; i.e. the existence of what we have named the frequentist posterior. On the other hand, it is the step-by-step density estimators, or predictives, $p(x_{m+1}|x_{1:m})$, which provide the accuracy.

After the data are exhausted, it is the $x_{\mathrm{mis}}$ which creates the uncertainty; rather than the notion of an alternative dataset of size n that could have been seen in the Frequentist approach. Simulating this $x_{\mathrm{mis}}$ from the predictive to form $x_{\mathrm{comp}}=(x_{\mathrm{mis}},x_{\mathrm{obs}})$ generates random outcomes of parameter values using the estimate $\theta (x_{\mathrm{comp}})$, and these form the basis of the frequentist posterior distribution. Each sampled $\theta_{\infty }$ represents a potentially true value and the collection quantifies the associated uncertainty.

For readers remaining unclear on the ideas, let us return to the standard Bayesian approach understood from the missing data perspective. Given $x_{1:n}$ the Bayesian derives what is regarded as the ‘best’ density estimator for $x_{n+1}$ via the posterior predictive (1.2). This is sampled, the model is updated using the sampled value and the procedure moves on to $x_{n+2}$, and so on. In the limit, the object of interest, let us assume it is θ, constructed from $x_{1:\infty }$, is a sample from the posterior based on the sample of size n. The key is the martingale sequence of estimators of θ as the samples are obtained.

The extended view of this strategy promoted in the paper is what exactly is the best density estimator for $x_{m+1}$ given $x_{1:m}$ for any m. Many density estimators are available. However, the Bayesian procedure tells us how to perform posterior analysis using any density estimator, including classical ones, such as those based on maximum-likelihood estimators.

## Data Availability

This article has no additional data.
